# Genetic counselling legislation and practice in cancer in EU Member States

**DOI:** 10.1093/eurpub/ckae093

**Published:** 2024-06-21

**Authors:** J Matt McCrary, Els Van Valckenborgh, Hélène A Poirel, Robin de Putter, Jeroen van Rooij, Denis Horgan, Marie-Luise Dierks, Olga Antonova, Joan Brunet, Adela Chirita-Emandi, Chrystelle Colas, Miriam Dalmas, Hans Ehrencrona, Claire Grima, Ramūnas Janavičius, Barbara Klink, Katalin Koczok, Mateja Krajc, Baiba Lace, Liis Leitsalu, Martin Mistrik, Milena Paneque, Dragan Primorac, Katharina M Roetzer, Joelle Ronez, Lucie Slámová, Elena Spanou, Kostas Stamatopoulos, Tomasz Stoklosa, Sonja Strang-Karlsson, Katalin Szakszon, Krzysztof Szczałuba, Jacqueline Turner, Marieke F van Dooren, Wendy A G van Zelst-Stams, Loredana-Maria Vassallo, Karin A W Wadt, Tamara Žigman, Tim Ripperger, Maurizio Genuardi, Marc Van den Bulcke, Anke Katharina Bergmann

**Affiliations:** Department of Human Genetics, Hannover Medical School, Hannover, Germany; Cancer Centre, , Department of Epidemiology and Public Health, Sciensano, Brussels, Belgium; Cancer Centre, , Department of Epidemiology and Public Health, Sciensano, Brussels, Belgium; Center for Medical Genetics, Ghent University Hospital, Ghent, Belgium; Department of Internal Medicine, Erasmus Medical Center, Rotterdam, The Netherlands; European Alliance for Personalised Medicine, Brussels, Belgium; Institute for Epidemiology, Social Medicine, and Health System Research, Hannover Medical School, Hannover, Germany; Department of Medical Genetics, Medical University of Sofia, Sofia, Bulgaria; Hereditary Cancer Program, Catalan Institute of Oncology, IDIBGI, Girona, Spain; Department of Microscopic Morphology, Genetics Discipline, Center of Genomic Medicine, University of Medicine and Pharmacy “Victor Babes”, Timisoara, Romania; Regional Center of Medical Genetics Timis, Clinical Emergency Hospital for Children “Louis Turcanu”, part of ERN ITHACA, Timisoara, Romania; Département de Génétique, Institut Curie, Paris, France; INSERM U830, Université Paris Cité, Paris, France; Ministry for Health, Valleta, Malta; Department of Clinical Genetics, Pathology and Molecular Diagnostics, Office for Medical Services, Region Skane, Lund, Sweden; Division of Clinical Genetics, Department of Laboratory Medicine, Lund University, Lund, Sweden; Mater Dei Hospital, Msida, Malta; Faculty of Medicine, Department of Human and Medical Genetics, Institute of Biomedical Sciences, Vilnius University, Vilnius, Lithuania; State Research Institute Center for Innovative Medicine, Vilnius, Lithuania; National Center of Genetics, Laboratoire National de Santé, Dudelange, Luxembourg; Department of Laboratory Medicine, University of Debrecen Medical and Health Science Center, Debrecen, Hungary; Institute of Oncology Ljubljana, Ljubljana, Slovenia; Riga East Clinical University, Riga, Latvia; Institute of Clinical and Preventive Medicine, University of Latvia, Riga, Latvia; Institute of Genomics, Faculty of Science and Technology, University of Tartu, Tartu, Estonia; Genetics and Personalized Medicine Clinic, Tartu University Hospital, Tartu, Estonia; Department of Medical Genetics, Unilabs, Spišská Nová Ves, Slovakia; CGPP—Centre for Predictive and Preventive Genetics, Institute for Molecular and Cell Biology (IBMC), Institute for Research and Innovation in Health (i3S), University of Porto, Porto, Portugal; ICBAS—School of Medicine and Biomedical Sciences, University of Porto, Porto, Portugal; St Catherine Specialty Hospital, Zagreb, Croatia; Medical School, University of Split, Split, Croatia; Faculty of Medicine, Josip Juraj Strossmayer University of Osijek, Osijek, Croatia; Faculty of Dental Medicine and Health, Josip Juraj Strossmayer University of Osijek, Osijek, Croatia; Medical School, University of Rijeka, Rijeka, Croatia; Medical School REGIOMED, Coburg, Germany; Eberly College of Science, The Pennsylvania State University, University Park, PA, USA; The Henry C. Lee College of Criminal Justice and Forensic Sciences, University of New Haven, West Haven, CT, USA; Department of Paediatrics, University Hospital Center Zagreb and University of Zagreb School of Medicine, Zagreb, Croatia; Labdia Labordiagnostik, Vienna, Austria; St Anna Children's Cancer Research Institute (CCRI), Vienna, Austria; Department of Human Genetics, Hannover Medical School, Hannover, Germany; Institute of Hematology and Blood Transfusion, Prague, Czech Republic; Clinical Genetics Department, The Cyprus Institute of Neurology and Genetics, Nicosia, Cyprus; Institute of Applied Biosciences, Centre for Research and Technology Hellas, Thessaloniki, Greece; Department of Tumor Biology and Genetics, Medical University of Warsaw, Warsaw, Poland; Department of Clinical Genetics, HUS Diagnostic Center, University of Helsinki and Helsinki University Hospital, Helsinki, Finland; Institute of Pediatrics, Faculty of Medicine, University of Debrecen, Debrecen, Hungary; Department of Medical Genetics, Medical University of Warsaw, Warsaw, Poland; Clinical Genetics Centre for Ophthalmology, The Mater Misericordiae University Hospital, Dublin, Ireland; Department of Clinical Genetics, Erasmus Medical Center, Rotterdam, The Netherlands; Department of Human Genetics, Radboud University Medical Center, Nijmegen, The Netherlands; State Research Institute Center for Innovative Medicine, Vilnius, Lithuania; Department of Clinical Genetics, University Hospital of Copenhagen, Rigshospitalet, Copenhagen, Denmark; Department of Clinical Medicine, Faculty of Health and Medical Sciences, University of Copenhagen, Copenhagen, Denmark; Department of Paediatrics, University Hospital Center Zagreb and University of Zagreb School of Medicine, Zagreb, Croatia; Department of Human Genetics, Hannover Medical School, Hannover, Germany; Sezione di Medicina Genomica, Dipartimento di Scienze della Vita e Sanità Pubblica, Università Cattolica del Sacro Cuore, Rome, Italy; UOC Genetica Medica, Dipartimento di Scienze di Laboratorio e Infettivologiche, Fondazione Policlinico Universitario A. Gemelli IRCCS, Rome, Italy; Cancer Centre, , Department of Epidemiology and Public Health, Sciensano, Brussels, Belgium; Department of Human Genetics, Hannover Medical School, Hannover, Germany

## Abstract

**Background:**

Somatic and germline genetic alterations are significant drivers of cancer. Increasing integration of new technologies which profile these alterations requires timely, equitable and high-quality genetic counselling to facilitate accurate diagnoses and informed decision-making by patients and their families in preventive and clinical settings. This article aims to provide an overview of genetic counselling legislation and practice across European Union (EU) Member States to serve as a foundation for future European recommendations and action.

**Methods:**

National legislative databases of all 27 Member States were searched using terms relevant to genetic counselling, translated as appropriate. Interviews with relevant experts from each Member State were conducted to validate legislative search results and provide detailed insights into genetic counselling practice in each country.

**Results:**

Genetic counselling is included in national legislative documents of 22 of 27 Member States, with substantial variation in legal mechanisms and prescribed details (i.e. the ‘who, what, when and where’ of counselling). Practice is similarly varied. Workforce capacity (25 of 27 Member States) and genetic literacy (all Member States) were common reported barriers. Recognition and/or better integration of genetic counsellors and updated legislation and were most commonly noted as the ‘most important change’ which would improve practice.

**Conclusions:**

This review highlights substantial variability in genetic counselling across EU Member States, as well as common barriers notwithstanding this variation. Future recommendations and action should focus on addressing literacy and capacity challenges through legislative, regulatory and/or strategic approaches at EU, national, regional and/or local levels.

## Introduction

Approximately 5–15% of cancers develop in the context of a cancer predisposition syndrome (CPS) caused by germline genetic variants.^1^ In the 27 European Union (EU) Member States alone, this manifests as an estimated incidence of 130 000–390 000 CPS-associated cancer diagnoses every year.^2^ New technologies capable of rapidly profiling these genetic alterations in both malignant cells (i.e. somatic testing) and non-diseased cells (i.e. germline testing) are a major asset for personalized diagnosis and prognosis, as well as targeted therapy and prevention programs promising improved outcomes in both index patients and their relatives.^3^

Paramount to the effective integration of genetic and genomic medicine into preventive and clinical cancer care is genetic counselling, defined succinctly by the National Society of Genetic Counselors (*US-based, but definition also commonly adopted internationally*^4^) as ‘the process of helping people understand and adapt to the medical, psychological and familial implications of [germline] genetic contributions to disease’.^5^ Genetic counselling is generally conducted both before and after germline genetic testing and includes: (1) collection and analysis of medical reports and family history to evaluate probabilities of hereditary diseases; (2) non-directive counselling regarding the potential consequences of genetic testing for prevention and treatment strategies, as well as for relatives and (3) information regarding preventive, treatment and surveillance options, as well as adaptations to the condition or risk revealed in genetic test results.^5^

Low-quality genetic counseling, including but not limited to counselling delivered by practitioners without appropriate genetics expertise, adversely impacts care. Specifically, low-quality counselling leads to inaccurate diagnoses, suboptimal clinical management (e.g. *with respect to early detection, treatment decisions and risk reducing strategies*), avoidable costs (e.g. *of unnecessary genetic tests*) and negative psychosocial outcomes in patients and family members.^6^ Considerable variation in genetic counselling practice across the EU has been previously described by the EuroGenTest consortium active from 2005 to 2009.^7^^,^^8^ A general shortage of clinical/medical genetics expertise across EU Member States has been reported as recently as 2020.^4^^,^^9^ Accordingly, new strategies are needed to ensure the equitable and sustainable delivery of high-quality genetic services by appropriately qualified experts; the need for these new strategies is particularly urgent in the context of increasing demands for genetic services linked to the push towards personalized medicine.^10^

This project, conducted as part of the European Commission-funded CAN.HEAL consortium (‘*Building the EU cancer and public health genomics platform*’; https://canheal.eu), aims to review genetic counselling legislation, regulations and current practice in EU Member States to provide a foundation for future European recommendations and action.

## Methods

This review was conducted in two phases, described sequentially below: (1) review of genetic counselling legislation in EU Member States and (2) semi-structured interviews^11^ with expert representatives in each EU Member State regarding current genetic counselling practice.

### Phase 1: review of genetic counselling legislation—search strategy and selection criteria

National legislative databases for each EU Member State (Supplementary table S1) were searched from 3 March 2023 to 10 March 2023 using relevant search terms: ‘genetic* AND counsel*’; ‘genetic* OR counsel*’. Search terms were translated as needed into local languages using freely available online translators DeepL and, if necessary due to unavailable language support in DeepL, GoogleTranslate. Publicly accessible national legislative databases were sourced from a European Commission Joint Research Centre report regarding general genomics legislation across the EU.^12^

Relevant national legislation and regulations were identified through a screening of titles from search results in each national database, followed by full-text review of all potentially relevant legislation and regulations. For the purposes of this review, ‘legislation’ is defined as a law passed by a national law-making body (e.g. *national parliament*), while ‘regulation’ refers to a legally binding rule enacted by a national government authority (e.g. *national health ministry*). Documents providing advice regarding genetic counselling but coming from non-government sources (e.g. institutional clinical practice guidelines) were excluded. When needed, legislation and regulations were translated into English for full-text review using the Translate Document feature in Microsoft Word 365 (Microsoft Inc.; Redmond, Washington). Legislation and regulations were included in the review if it references, mentions, describes and/or impacts genetic counselling in a clinical and/or preventive cancer context, defined according to the National Society of Genetic Counselors definition included in the Introduction section.^5^

### Phase 2: interview survey on genetic counselling practice

Individuals from each EU Member State with experience and expertise in genetic counselling participated in a 10–30-min semi-structured interview with the lead author (J.M.M.). Guiding interview questions are outlined in Supplementary table S2. Interviews were conducted *via* video or telephone calls, with the exception of communication with Belgian representatives for whom responses to interview questions were delivered *via* email. Interview questions were developed in consultation with a focus group of expert clinical and molecular geneticists, bioinformaticians and oncologists within the activities of the CAN.HEAL consortium. Additionally, responses to two follow-up questions were collected by e-mail after the interview: (1) what is the most important change which could be (or is planning to be) made to improve genetic counselling practice in <country>?; (2) Apart from insurance reimbursement and health/genetic literacy, are there any other significant barriers to patient access to genetic counselling in <country>?

Interview participants were primarily sourced from the CAN.HEAL consortium, comprising representation from 16 of 27 Member States, and second-degree contacts of consortium members. Interview participants were selected based on their experience with clinical genetic services in their Member State, as demonstrated by publication records and their current professional role. Given the variation in practice across EU Member States, no restrictions were placed on the specialization of interview participants—the majority of interview participants were clinical/medical geneticists or genetic counsellors (*Master’s degree qualified allied health professionals*) but non-genetics physicians and health professionals with genetic counselling expertise/experience were also interviewed where appropriate. ‘Medical geneticists’ and ‘clinical geneticists’ are functionally equivalent titles used in different Member States to describe physicians specialized in genetics.

### Data analysis

Legislation, regulation and interview results regarding current practice were grouped around four key themes defined *a priori*: who, what, when and where/how. Information related to insurance reimbursement barriers were originally classed into the ‘when’ category but were judged to be substantially different during analysis and given their own heading in results reporting.

### Ethical approval

This is a collaborative project in which all contributors (i.e. interview participants) are named co-authors. Accordingly, ethical approval was not required.

## Results

### International agreements & EU regulation

A majority of Member States (22 of 27) are signatories to the Convention on Human Rights and Biomedicine—commonly known as the ‘Oviedo Convention’ (1997)—although this binding treaty has been ratified without reservations in only 14 of 27 countries (see table 1). The Oviedo Convention makes brief specific reference to genetic counselling in Article 12, Predictive Genetic Tests, stating that such tests are ‘subject to appropriate genetic counselling’.^13^ An Additional Protocol to the Oviedo Convention (2008) concerning Genetic Testing for Health Purposes has been signed by six Member States, although only ratified by three (table 1). Article 8 of the Additional Protocol—Information and Genetic Counselling—expands on Article 12 of the Oviedo Convention by further specifying the types of predictive tests ‘subject to appropriate counselling’, adding that all genetic tests should be preceded by ‘appropriate information’ regarding their ‘purpose…nature…[and] implications’, and affirming that ‘genetic counselling shall be given in a non-directive manner’.^14^

**Table 1:** Signatures of the Oviedo Convention and its additional Genetic Testing Protocol and national legislation (*laws passed by parliament*) related to genetic counselling in EU Member States.

	Signatory to Oviedo Convention			Defined in national legislation?		
Country	General Convention (1997)	Genetic Testing Protocol (2008)	Additional National Legislation	Who can provide genetic counselling	What must be discussed during genetic counselling	When must genetic counselling be provided
Austria	–	–	Gene Technology Act (1994)	Yes	Yes	Yes
Belgium	–	–	Law 14/7/1994 Compulsory Health Care and Indemnity Insurance Act, Article 22, 18° *Implemented by*: Agreement between the medical care insurance committee and the centres for human heredity for benefits in kind concerning genetic disorders: Genetic counselling, DNA tests carried out abroad (2011)	Yes	Yes	Yes
Bulgaria	Yes	–	Health Law (2005)	–	–	–
Croatia	Yes^a^	–	Law on the Protection of Patients’ Rights (2008)	–	–	–
Cyprus	Yes	–	–	–	–	–
Czech Republic	Yes	Yes	Act 373/2011 Coll. on Specific Health Care Services	Yes	Yes	Yes
Denmark	Yes^b^	–	Amendment of the Healthcare Law, 27/01/2022	–	–	–
Estonia	Yes	–	–	–	–	–
Finland	Yes	Yes^c^	–	–	–	–
France	Yes^a^	Yes^c^	Law no. 2021-1017 of August 2, 2021 on Bioethics	Yes	Yes	Yes
Germany	–	–	Gene Diagnostics Law (2009)	Yes	Yes	Yes
Greece	Yes	–	–	–	–	–
Hungary	Yes	–	Parliamentary Act No. XXI (2008)	Yes	Yes	Yes
Ireland	–	–	Disability Act (2005)	–	Yes	Yes
Italy	Yes^c^	–	–	–	–	–
Latvia	Yes	–	Human Genome Research Law (2004)	–	–	–
Lithuania	Yes	–	–	–	–	–
Luxembourg	Yes^-^	Yes^c^	Nomenclature Law (2014)	Yes	Yes	
			Hospital Law (2018)	–	–	–
Malta	–	–	–	–	–	–
Netherlands	Yes^c^	–	Special Medical Procedures Act (1997)	Yes	–	–
Poland	Yes^c^	–	–	–	–	–
Portugal	Yes	Yes	Personal Genetic Information and Health Information Act (2005)	Yes	–	Yes
Romania	Yes	–	–	–	–	–
Slovakia	Yes	–	–	–	–	–
Slovenia	Yes	Yes	Criminal Code	–	–	Yes
Spain	Yes	–	Law 14/2007, of 3 July, on Biomedical Research	–	Yes	Yes
Sweden	Yes^c^	–	Genetic Integrity Act (2006)	–	–	–

See Supplementary table S2 for further details regarding specific sections of national legislation judged to address ‘who’, ‘what’ and ‘when’ considerations.

a Signed with reservations.

b Signed with reservations, declarations/denunciations/derogations and territorial application.

c Signed but not ratified.

Further, genetic counselling in all EU Member States is subject to the provisions of EU Regulation 2017/746 on *in vitro* diagnostic medical devices. Article 4 of this Regulation—Genetic information, counselling and informed consent—stipulates that all clinical genetic tests must be accompanied by information regarding ‘the nature, the significance and the implications of the genetic test, as appropriate’.^15^

### National legislation and regulation

**Figure 1:**
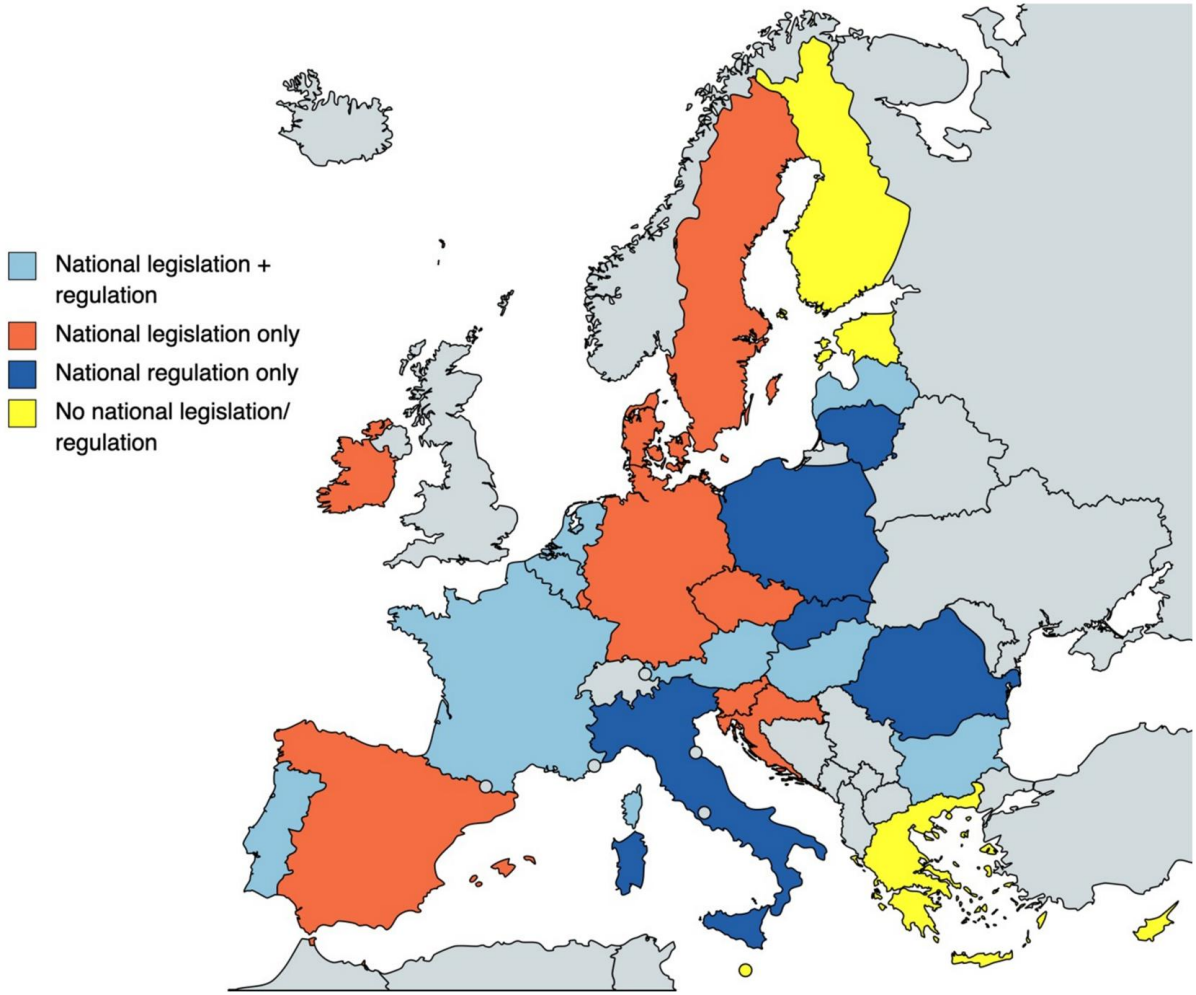
Overview of diverse landscape of genetic counselling legislation (*laws passed by parliament*) and regulation (*legally binding rules enacted by a national government authority*) in EU Member States. Figure created with mapchart.net, used with permission.

A total of 18 pieces of national legislation (*laws passed by parliament*) addressing genetic counselling are present in 17 of 27 Member States. Nineteen national regulations (*legally binding rules enacted by a national government authority*) are currently active in 13 Member States. A combination of legislation and regulation is active in eight Member States. No national legislation or regulations addressing genetic counselling are present in 5 of 27 Member States. See figure 1 and tables 1 and 2 for further details.

**Table 2:** Regulations (*legally binding rules enacted by a national government authority*) addressing genetic counselling in EU Member States.

		Defined in National Regulation?		
Country	National Regulation	Who can provide genetic counselling	What must be discussed during genetic counselling	When must genetic counselling be provided
Austria	Quality Standard for Genetic Counselling and Diagnostics (2015)	Yes	Yes	Yes
Belgium	Royal Decree establishing the standards to be met by centres for human genetics (1987)	–	Yes	–
	Ministerial Decree amending the Ministerial Decree of 23 of May 2017 establishing special criteria for recognition of physician specialists, internship masters and internship services in clinical genetics (2018)	Yes	Yes	–
Bulgaria	Medical Standards for Medical Genetics (2010)	Yes	Yes	Yes
Croatia	–	–	–	–
Cyprus	–	–	–	–
Czech Republic	–	–	–	–
Denmark	–	–	–	–
Estonia	–	–	–	–
Finland	–	–	–	–
France	Order of 27 May 2013 defining the rules of good practice applicable to the examination of a person’s genetic characteristics for medical purposes	Yes	Yes	Yes
	Decree no. 2022-1488 of November 29, 2022 on the conditions for prescribing certain medical biology examinations and communicating their results by genetic counsellors	Yes	–	–
Germany	–	–	–	–
Greece	–	–	–	–
Hungary	Directive of the Ministry of Human Resources on genetic counselling, ID: 002092 (2020)	Yes	Yes	Yes
Ireland	–	–	–	–
Italy	Guidelines for Medical Genetics (2004)	Yes	Yes	Yes
	General Authorisation No. 8/2014 for the Processing of Genetic Data (updated 2016)	Yes^a^	Yes	Yes
Latvia	Regulation No. 555, Adopted 28 August 2018—Procedures for the Organization of and Payment for Health Care Service under Medical Treatment Law (1997)	–	–	Yes
Lithuania	Order of the Ministry of Health V-745 (2012)	Yes	–	–
	Order of the Ministry of Health V-1458 (2014)	Yes	–	Yes
Luxembourg	–	–	–	–
Malta	–	–	–	–
Netherlands	Planning Decision on Clinical Genetic Research and Genetic Counselling (1998)	Yes	–	–
Poland	Order of the Minister of Health of 21st July 2022 amending the Regulation on guaranteed benefits in the field of ambulatory care for families with high risk of hereditary cancer	Yes	Yes	Yes
Portugal	Order of the Ministry of Health #5411/97	Yes	–	–
Romania	Establishment of the Medical Genetics Network (2014)	Yes	–	–
	Order no. 774 of September 18, 2023	–	–	–
	Appendices no. 1 and 2 to Government Decision no. 521/2023 for approval of the service packages and the framework contract that regulates the conditions for the provision of medical assistance, medicines and medical devices, within the social health insurance system	Yes	Yes	–
Slovakia	The Concept of Healthcare in the Field of Medical Genetics (2014)	Yes	Yes	Yes
Slovenia	Clinical pathway and scope of work of a genetic consultant in the process of treating a patient in the Oncology Genetic Counselling and Testing Clinic^b^	Yes	Yes	Yes
Spain	–	–	–	–
Sweden	–	–	–	–

See Supplementary table S3 for further details regarding specific sections of national regulations judged to address ‘who’, ‘what’ and ‘when’ considerations.

a Specifies that genetic counselling must be provided by ‘genetic consultants’ but does not specify the training or specialization needed to qualify for this distinction.

b Legally binding clinical pathway issued by the Oncology Institute Ljubjana, a non-government source, but included in this table for reference due to its legal weight.

Substantial variation exists in the content of national legislation and regulations, in particular regarding their coverage of the ‘who’, ‘what’ and ‘when’ of genetic counselling (tables 1 and 2). Legislation and regulations in Belgium,^16^ Luxembourg^17^ and the Netherlands^18^ also address ‘where’ genetic counselling can be performed, restricting its delivery to defined centres of medical genetics expertise (Supplementary tables S3 and S4). Regulations in Bulgaria^19^ and Lithuania^20^ specify the physical/design requirements for clinics where genetic counselling can be performed (Supplementary table S4).

### Practice

A total of *N* = 35 individuals with expertise and experience in genetic counselling from each of the 27 EU Member States contributed to interviews related to genetic counselling practice in their country. Of these individuals, *N* = 22 were physicians specialized in clinical/medical genetics (mean age = 48 years, range 37–66; mean years of experience = 18, range 3–36), *N* = 6 were genetic counsellors (mean age = 43, range 31–55; mean years of experience = 16, range 2–30), *N* = 5 were practicing physicians from non-genetic specialties (oncology, haematology, immunology, paediatrics) (mean age = 54, range 42–58; mean years of genetics experience = 24, range 8–30) and *N* = 2 were from other health specialties (genetic epidemiology, public health medicine) (mean age = 48, range 36–59; mean years of genetics experience = 17, range 6–27).

Consistent with variation in national legislation and regulation, substantial variation in genetic counselling practice between countries was reported. Clinical/medical genetics—used interchangeably in different countries—is a recognized specialty in 26 of 27 Member States. Clinical/medical geneticists are exclusively able to order germline genetic tests and deliver genetic counselling in three Member States. Clinical/medical geneticists exclusively deliver genetic counselling in a further three Member States. Genetic counselling is provided by varied combinations of clinical/medical geneticists, non-geneticist physicians, nurses (*with and without specialized genetics training*) and genetic counsellors in the remaining 21 Member States. Genetic counsellors are only officially recognized within the health system in France but are active and integrated into the provision of genetic services in a further six Member States.

Delivery of both pre- and post-test counselling is mandatory in 12 Member States and is typically provided at both time points in 13 additional Member States. Telemedical genetic counselling is currently legally possible in 25 of 27 Member States but only frequently utilized in eight Member States. See Supplementary table S5 for full details.

### Barriers to genetic counselling—genetic literacy

The genetic literacy of patients and non-geneticist physicians was reported as an ongoing challenge to effective genetic counselling delivery in all (27 of 27) EU Member States. Initiatives and resources to increase the genetic literacy of patients and provide continuing education/training for non-geneticist clinicians are disparate across Member States. These resources and initiatives are primarily organized at the local and regional level, as well as by individual clinicians. National genetic literacy initiatives were reported as being present in a minority of Member States (7 of 27). Official mandates to provide genetic education to the general population and/or non-geneticist health professionals are included in regulations of four Member States (Supplementary table S3).^18^^,^^21–23^

### Barriers to genetic counselling—workforce capacity, insurance reimbursement and others

The capacity of the current workforce to deliver timely genetic counselling by expert personnel was reported as a barrier to genetic counselling practice by representatives from 25 of 27 Member States. Insurance reimbursement was reported as a barrier to genetic counselling practice in 56% (15 of 27) of Member States (Supplementary table S5). Other reported barriers to counselling were: socioemotional issues (e.g. discrimination, emotional distress and personal fear); scepticism from non-genetics physicians and institutions regarding the utility of genetics in patient care; an inability to directly contact potentially at-risk family members of patients; over-‘democratization’ of genetic counselling to non-genetics physicians and allied health professionals resulting in low-quality counselling; and regional inequalities.

### Most important change to improve genetic counselling

Common ‘most important changes’ noted by multiple (i.e. ≥2) Member State representatives were (table 3): recognition and/or integration of genetic counsellors (10 of 27 Member States); updated and/or additional genetic counselling legislation/regulation (six Member States); mainstreaming of some genetic counselling to non-genetics medical professionals (three Member States); the recognition of a clinical genetics specialty (two Member States); increased numbers of clinical/medical geneticists (two Member States) and education initiatives for non-genetics medical professionals (two Member States).

**Table 3:** Perspectives from representatives from each EU Member State regarding the ‘most important’ change that could/will be made to improve genetic counselling in their country.

Member State	Most important change (upcoming/desired)
Austria	Increased genetics capacity (additional clinical geneticists and/or integration of genetic counsellors)
Belgium	Recognition of genetic counsellors as a healthcare profession
Bulgaria	Legislation/regulation to standardize genetic counselling practice
Croatia	Specialization in clinical genetics (*upcoming; first residents in 2023*)
Cyprus	Legislation/regulation to standardize genetic counselling practice
Czech Republic	Improved genetics education for non-genetics medical professionals
Denmark	More systemized adult cancer predisposition outpatient clinics
Estonia	Recognition of genetic counsellors as a healthcare profession & establishment of a national training program to assist with their integration
Finland	Increased genetics capacity and regulation to ensure genetic counselling is provided by adequately qualified professionals in the context of direct-to-consumer tests
France	Improved access to genetic counselling throughout France through a network of partner and referral clinics
Germany	Re-organization of the health system to better integrate genetic services into care pathways
Greece	Improved government policies and a clear framework regarding clinical/laboratory genetics specialties
Hungary	Increased support for genome wide testing, arrays & exomes from health insurance (*i.e. increased testing options to support better counselling*)
Ireland	Increased mainstreaming of genetic counselling (*planned)*
Italy	Recognition of genetic counsellors as a healthcare profession
Latvia	National regulation to standardize requirements of genetic service practice
Lithuania	Expansion of indications for genetic counselling
Luxembourg	Increased genetics capacity through recruitment of additional clinical geneticists and/or recognition and integration of genetic counsellors as a healthcare profession
Malta	Recognition and integration of genetic counsellors (*known as ‘Genomic Care Coordinators’*) as a healthcare profession (*in process*)
Netherlands	Implementation of mainstreaming and telemedicine to meet increasing demands and ensure accessibility. Implementation of genome sequencing as a first-tier test for patients with suspected rare genetic diseases
Poland	Introduction of genetic counsellors (*planned for 2024*)
Portugal	Recognition and integration of genetic counsellors as a healthcare profession
Romania	Improved genetic counselling regulation (*in process*) and introduction of adult cancer predisposition genetic testing *(in process)*
Slovakia	Increased genetics capacity (*additional medical geneticists—planned; integration of genetic nurses—in discussion*)
Slovenia	Integration of genetic/genomic counsellors *(nurses with special training; in process)*
Spain	Legal recognition & integration of a clinical genetics specialty
Sweden	Improved genetics education for non-genetics medical professionals to facilitate mainstreaming

## Discussion

This manuscript reveals a diversity of approaches to both genetic counselling legislation/regulation and practice across EU Member States in the context of cancer. This diversity may denote a beneficial flexibility in genetic counselling practice across the EU, as culturally and contextually appropriate counselling delivery has been shown to be important to counselling quality.^24^^,^^25^ Similarly, a mosaic of genetic counselling legislation/regulations across Member States enables genetic counselling to be positioned within specific local, regional and national approaches to genetic testing, data structures and security, research and broader healthcare practice with varied organizational concepts (e.g. ‘top-down’ vs. ‘bottom-up’ approaches to healthcare implementation^26^).

However, common workforce capacity and health/genetic literacy challenges across EU Member States highlight the need for new strategies to ensure equitable and timely delivery of high-quality genetic counselling by appropriately qualified experts. Common perspectives across Member States regarding the ‘most important changes’ to improve genetic counselling—recognition/integration of genetic counsellors; updated legislation/regulation—emphasize opportunities for synergistic action.

With respect to workforce capacity challenges noted by 25 of 27 Member States, national and regional ‘use cases’ from Sweden and Catalonia, Spain present two distinct approaches which could be instructive for other Member States. In Sweden, pre-test counselling and ordering of genetic tests for common indications outlined in national guidelines has been mainstreamed to other specialties—e.g. surgeons in hereditary breast cancer. Patients are typically referred to clinical genetics departments for post-test counselling only in case of significant findings, but any patient who actively requests contact with genetics professionals for pre-test counselling or post-test counselling after a negative laboratory investigation will also be referred. Accordingly, the burden of counselling for most cases has been shifted outside of clinical genetics departments without significant impacts on quality. Ongoing continuing education initiatives (e.g. *seminars, diploma & degree courses*) are tasked with improving the genetic competencies of non-genetics physicians and allied health professionals. Additional improvements in this domain are noted to still be required to further improve counselling quality (table 3).

In Spain, clinical genetics is not a recognized medical specialty despite the inclusion of clinical genetics activities in national legislation. Accordingly, to address both capacity and expertise constraints, the regional health system of Catalonia has hired approximately 20 genetic counsellors (*c. 2.7 per 1 000 000 population*). These genetic counsellors are Master’s degree qualified and recognized at the institutional level within Catalonia but not within the broader Spanish health system. It is particularly interesting to note that both use cases have addressed capacity challenges outside of national genetic counselling legislation or regulation. Rather, these initiatives have been facilitated by national genetic testing guidelines and institution-level accreditations.

The caveat to these ‘use cases’, however, is that the degree to which these approaches can be replicated in other regions and Member States is unclear. An ongoing study piloting the mainstreaming of genetic counselling to oncologists in Germany has found the capacity and willingness of oncologists to deliver counselling to be a significant barrier, even after receiving targeted genetics education.^27^ Capacity gains from mainstreaming must also be balanced with potential reductions in genetic counselling quality, reported both by prior research^6^ and multiple national experts interviewed for this project. A general and increasing shortage of both clinical geneticists^9^ and broader health professionals^28^ is likely to preclude the resolution of capacity challenges solely through the hiring of additional clinical geneticists and/or genetic counsellors in many contexts. Similarly, the mainstreaming of genetic counselling to other physicians and/or health professionals must be done judiciously to avoid simply re-locating capacity challenges.

Poor integration and a lack of recognition of genetic counsellors in almost all EU Member States restrict the present ability of these allied health professionals to address workforce challenges. Better integration of the 450+ existing genetic counsellors in the EU^4^ thus presents an initial ‘low-hanging fruit’ solution to workforce capacity challenges. Whether this integration also involves formal recognition through national legislation/regulation should be discussed within each national context. National legal recognition would guarantee educational standards and likely improve the attractiveness of the profession to future students.^4^ However, such national recognition was noted by some national representatives to necessitate a disproportionately burdensome development of national regulatory and educational infrastructure. Several existing elements provide a foundation for a potential European solution to the education and regulation of genetic counsellors: established Master’s degree programs in several EU Member States; registration protocols for genetic counsellors with the European Board of Medical Genetics and inclusion of ‘collaboration with Genetic Counsellors’ as a key clinical skill in the latest EU of Medical Specialists training standards for medical genetics physicians.^29^

Telemedical genetic counselling promises to provide a more efficient alternative to face-to-face counselling and thus a potential means of addressing some workforce capacity challenges.^30^ However, despite being legally possible in 25 Member States, telemedical counselling was only reported as being conducted frequently in eight Member States. Reservations from national representatives were primarily related to perceived reductions in quality compared to face-to-face consultations. In particular, reservations regarding telemedical counselling were noted for complex clinical scenarios and the return of positive/significant test results, when telemedical consults are not geographically necessary, and where reimbursement for telemedical counselling is challenging or nonexistent. Innovations which increase genetic counselling efficiency—e.g. digital tools which independently capture family health history, a particularly time-consuming component of genetic counselling^31^^,^^32^—may provide additional solutions with potential EU-wide applications.

Adjacent to workforce capacity challenges, national initiatives aiming to increase the genetic literacy of patients/citizens and/or provide continuing genetics education to non-genetics health professionals were only reported in a minority of Member States. This is a notable absence of focused resources given the links between genetic literacy and the effectiveness of genetic counselling and genetic services,^33^^,^^34^ as well as the acceptance and uptake of genetic services more broadly.^35^ However, genetics training and education for health professionals and the broader population have been given a mandate through two respective ‘Triplets of Action’ in the Strategic Research and Innovation Agenda for Personalized Medicine published in April 2023 by the European Commission-backed European Partnership for Personalized Medicine.^35^ Near-future discussion and consensus action will be key to the effective implementation of this mandate across EU Member States; e.g. through the development of consensus recommendations planned as part of the European Commission-funded CAN.HEAL Consortium.

The role of regulation and legislation in facilitating equitable and timely access to high-quality counselling also merits discussion, particularly given that a majority of ‘most important changes’ noted by Member State representatives relate to prospective legislative/regulatory changes (e.g. *recognition of genetic counsellors/clinical geneticists; further standardization of genetic counselling practice*). Additionally, regulation/legislation is likely the most effective means of addressing insurance barriers to counselling noted by 15 Member States. In each national context, particular attention must be paid to the selection of the legal instrument (i.e. legislation vs. regulation) that is the most appropriate for the intended permanence vs. flexibility, respectively, of the action in the context of rapidly changing knowledge and best practices. For example, recognition and integration of genetic counsellors and clinical geneticists is a long-term change which may be best enacted through legislation. Conversely, standardization of genetic counselling practice is likely to be continually impacted by revised knowledge and best practices and thus may be best achieved through regulation. Further, the potential role of EU-level action as an efficient means of addressing common challenges should be examined, albeit with particular attention to permanence vs. flexibility considerations.

Limitations of this review include our consultation with only one to three representatives from each Member State. These representatives were selected due to their expertise and experience in genetic counselling in their country but may not have been able to comment comprehensively on local and regional variations occurring within their national contexts. Additionally, this project was conceived and conducted in the context of genetic counselling for hereditary cancer predisposition in each Member State. Accordingly, while results may be applicable and relevant to other disease contexts, such translation should be performed with caution.

In conclusion, this review highlights substantial variability in genetic counselling practice and legislation/regulation across EU Member States. Common workforce capacity and health/genetic literacy challenges were prevalent across Member States notwithstanding this variation. Integration/recognition of genetic counsellors and updates to legislation/regulation were the most commonly noted ‘most important changes’ which could be made to improve access to and delivery of genetic counselling in the context of cancer. Future discussions and action should focus addressing literacy and capacity challenges through legislative, regulatory and/or strategic approaches to ensure equitable, timely and high-quality genetic counselling across Member States.

## Supplementary Material

ckae093_Supplementary_Data

## Data Availability

All study data can be found within the published article and its supplementary files.

## References

[1] Ding L , Bailey MH, Porta-Pardo E, et al Perspective on oncogenic processes at the end of the beginning of cancer genomics. Cell 2018;173:305–20.e10. 29625049 10.1016/j.cell.2018.03.033 PMC5916814

[2] Dyba T , Randi G, Bray F, et al The European cancer burden in 2020: incidence and mortality estimates for 40 countries and 25 major cancers. Eur J Cancer 2021;157:308–47. 34560371 10.1016/j.ejca.2021.07.039 PMC8568058

[3] Taber KAJ , Dickinson BD, Wilson M. The promise and challenges of next-generation genome sequencing for clinical care. JAMA Intern Med 2014;174:275–80. 24217348 10.1001/jamainternmed.2013.12048

[4] Abacan M , Alsubaie L, Barlow-Stewart K, et al The global state of the genetic counseling profession. Eur J Hum Genet 2019;27:183–97. 30291341 10.1038/s41431-018-0252-x PMC6336871

[5] Resta R , Biesecker BB, Bennett RL, et al A new definition of genetic counseling: National Society of Genetic Counselors’ task force report. J Genet Couns 2006;15:77–83. 16761103 10.1007/s10897-005-9014-3

[6] Bensend TA , Veach PM, Niendorf KB. What’s the harm? Genetic counselor perceptions of adverse effects of genetics service provision by non-genetics professionals. J Genet Couns 2014;23:48–63. 23754506 10.1007/s10897-013-9605-3

[7] Borry P , Nys H, Goffin T, Dierickx K. Genetic Testing and Counselling: European Guidance. Report No.: 9033465795. Katholieke Universiteit, Centre for Biomedical Ethics and Law, 2007.

[8] Rantanen E , Hietala M, Kristoffersson U, et al Regulations and practices of genetic counselling in 38 European countries: the perspective of national representatives. Eur J Hum Genet 2008;16:1208–16. 18478036 10.1038/ejhg.2008.93

[9] Dragojlovic N , Borle K, Kopac N, et al The composition and capacity of the clinical genetics workforce in high-income countries: a scoping review. Genet Med 2020;22:1437–49. 32576987 10.1038/s41436-020-0825-2

[10] Dragojlovic N , Kopac N, Borle K, et al Utilization and uptake of clinical genetics services in high-income countries: a scoping review. Health Policy 2021;125:877–87. 33962789 10.1016/j.healthpol.2021.04.010

[11] Adeoye‐Olatunde OA , Olenik NL. Research and scholarly methods: semi‐structured interviews. J Am Coll Clin Pharm 2021;4:1358–67.

[12] Angers A , Bohacova A, Kaye J, et al Overview of EU national legislation on genomics. 2018. Available at: https://op.europa.eu/en/publication-detail/-/publication/503c92f7-ff55-11e8-a96d-01aa75ed71a1 (7 March 2023, date last accessed).

[13] Council of Europe. Convention on Human Rights and Biomedicine. 1997. Available at: https://rm.coe.int/168007cf98 (3 March 2023, date last accessed).

[14] Council of Europe. Additional Protocol to the Convention on Human Rights and Biomedicine Concerning Genetic Testing for Health Purposes. 2008. Available at: https://rm.coe.int/1680084824 (3 March 2023, date last accessed). 19180986

[15] European Parliament. Regulation 2017/746 on In Vitro Diagnostic Medical Devices and Repealing Directive 98/79/EC and Commission Decision 2010/227/EU. 2017. Available at: https://eur-lex.europa.eu/legal-content/EN/TXT/PDF/?uri=CELEX:32017R0746 (30 June 2023, date last accessed).

[16] National Institute for Health and Disability Insurance (Belgium). Agreement Between the Medical Care Insurance Committee and the Centres for Human Heredity for Benefits in Kind Concerning Genetic Disorders: Genetic Counselling, DNA Tests Carried Out Abroad. 2011. Available at: https://www.google.com/url?sa=t&rct=j&q=&esrc=s&source=web&cd=&cad=rja&uact=8&ved=2ahUKEwjjhb-ew4aAAxXUXaQEHecsCucQFnoECA0QAw&url=https%3A%2F%2Fwww.college-genetics.be%2Fpdf%2FConventieGeneticsCounsellor.pdf&usg=AOvVaw3m-ExyIxVRhAjES-UTaWLQ&opi=89978449 (7 July 2023, date last accessed).

[17] Chamber of Deputies of Luxembourg. Hospital Law. 2018. Available at: https://www.legilux.public.lu/eli/etat/leg/loi/2018/03/08/a222/jo. (1 November 2023, date last accessed).

[18] Secretary of State for Health Welfare and Sport (Netherlands). Planning Decision on Clinical Genetic Research and Genetic Counselling. 1998. Available at: https://wetten.overheid.nl/BWBR0014594/2018-08-01 (4 May 2023, date last accessed).

[19] Ministry of Health (Bulgaria). Medical Standards for Medical Genetics. 2010. Available at: https://www.mh.government.bg/media/filer_public/2015/11/18/medicinska-genetika.pdf (23 June 2023, date last accessed).

[20] Ministry of Health (Lithuania). Order of the Ministry of Health V-745 (2012). 2012 (10 March 2023, date last accessed).

[21] Ministry of Health (Slovakia). The Concept of Healthcare in the Department of Medical Genetics. 2014. Available at: https://www.google.com/url?sa=i&rct=j&q=&esrc=s&source=web&cd=&ved=0CAIQw7AJahcKEwjo8_zo2Pf_AhUAAAAAHQAAAAAQAw&url=https%3A%2F%2Fwww.health.gov.sk%2FZdroje%3F%2FSources%2Fdokumenty%2Fvestniky_mz_sr%2F2014%2Fvestnik-21-22-2014.pdf&psig=AOvVaw3qpmyEymuaiTA8ZT3Bt5Sz&ust=1688650864404466&opi=89978449 (23 June 2023, date last accessed).

[22] Ministry of Health (Italy). Guidelines for Medical Genetics. 2004. Available at: https://www.gazzettaufficiale.it/eli/id/2004/09/23/04A09280/sg (19 April 2023, date last accessed).

[23] Ministry of Health (Romania). Establishment of the Medical Genetics Network. 2014. Available at: https://legislatie.just.ro/Public/DetaliiDocument/163135 (22 June 2023, date last accessed).

[24] Barlow-Stewart K , Yeo SS, Meiser B, et al Toward cultural competence in cancer genetic counseling and genetics education: lessons learned from Chinese-Australians. Genet Med 2006;8:24–32. 16418596 10.1097/01.gim.0000195884.86201.a0

[25] Allford A , Qureshi N, Barwell J, et al What hinders minority ethnic access to cancer genetics services and what may help? Eur J Hum Genet 2014;22:866–74. 24253862 10.1038/ejhg.2013.257 PMC4060110

[26] Stenzinger A , Moltzen EK, Winkler E, et al Implementation of precision medicine in healthcare – a European perspective. J Intern Med 2023;294:437–54. 37455247 10.1111/joim.13698

[27] Tecklenburg J , Vajen B, Morlot S, et al OnkoRiskNET: a multicenter, interdisciplinary, telemedicine-based model to improve care for patients with a genetic tumor risk syndrome. BMC Health Serv Res 2022;22:1–8. 35729592 10.1186/s12913-022-08172-2 PMC9210737

[28] World Health Organization. Global Strategy on Human Resources for Health: Workforce 2030. 2016.

[29] European Union of Medical Specialists. European Training Requirements for the Specialty of Medical Genetics. 2023. Available at: https://www.uems.eu/__data/assets/pdf_file/0007/47518/ETR_Clinical-Genetics_approved.pdf (7 October 2023, date last accessed).

[30] Otten E , Birnie E, Ranchor AV, van Langen IM. Telegenetics use in presymptomatic genetic counselling: patient evaluations on satisfaction and quality of care. Eur J Hum Genet 2016;24:513–20. 26173963 10.1038/ejhg.2015.164 PMC4929881

[31] Welch BM , Wiley K, Pflieger L, et al Review and comparison of electronic patient-facing family health history tools. J Genet Couns 2018;27:381–91. 29512060 10.1007/s10897-018-0235-7 PMC5861014

[32] Bucheit L , Johansen Taber K, Ready K. Validation of a digital identification tool for individuals at risk for hereditary cancer syndromes. Hered Cancer Clin Pract 2019;17:2–10. 30651894 10.1186/s13053-018-0099-8 PMC6330430

[33] van der Giessen J , Fransen MP, Spreeuwenberg P, et al Communication about breast cancer genetic counseling with patients with limited health literacy or a migrant background: evaluation of a training program for healthcare professionals. J Commun Genet 2021;12:91–9. 10.1007/s12687-020-00497-x PMC7846648 33319336

[34] Little ID , Koehly LM, Gunter C. Understanding changes in genetic literacy over time and in genetic research participants. Am J Human Genet 2022;109:2141–51. 36417915 10.1016/j.ajhg.2022.11.005 PMC9748356

[35] European Partnership for Personalised Medicine. The Strategic Research & Innovation Agenda (SRIA) for Personalised Medicine (PM). 2023. Available at: https://www.icpermed.eu/media/content/EPPerMed-SRIA.pdf (1 November 2023, date last accessed).

