# Supplementary material for: Genetic counselling legislation and practice in cancer in EU Member States
Source: Eur J Public Health. 2024 Jun 21;34(4):666–75. doi: 10.1093/eurpub/ckae093 (PMC11293827; doi:10.1093/eurpub/ckae093)
Supplement: ckae093_Supplementary_Data [file ckae093_supplementary_data.pdf]

Genetic counselling legislation and practice in cancer in EU Member States

Authors: J. Matt McCrary, Els Van Valckenborgh, Hélène A. Poirel, Robin de Putter, Jeroen van Rooij, Denis Horgan, Marie-Luise Dierks, Olga Antonova, MD, Joan Brunet, MD, Adela Chirita-Emandi, Chrystelle Colas, Miriam Dalmas, Hans Ehrencrona, Claire Grima, Ramūnas Janavičius, Barbara Klink, Katalin Koczok, Mateja Krajc, Baiba Lace, Liis Leitsalu, Martin Mistrik, Milena Panenque, Dragan Primorac, Katharina M. Roetzer, Joelle Ronez, Lucie Slámová, Elena Spanou, Kostas Stamatopoulos, Tomasz Stoklosa, Sonja Strang-Karlsson, Katalin Szakszon, Krzysztof Szczałuba, Jacqueline Turner, Marieke F. van Dooren, Wendy A.G. van Zelst-Stams, Loredana-Maria Vassalo, Karin A.W. Wadt, Tamara Žigman, Tim Ripperger, Maurizio Genuardi, Marc van den Bulcke, Anke Katharina Bergmann

Supplementary Tables

Table of Contents

Supplementary Table 1……………………………………………………………..*Page 2*

Supplementary Table 2……………………………………………………………..*Page 3*

Supplementary Table 3……………………………………………………………..*Page 4*

Supplementary Table 4……………………………………………………………..*Page 6*

Supplementary Table 5……………………………………………………………..*Page 10*

Member State	Legislation Database URL
Austria	https://www.ris.bka.gv.at/
Belgium	http://www.ejustice.just.fgov.be/cgi/welcome.pl
Bulgaria	https://www.lex.bg/
Croatia	https://narodne-novine.nn.hr/search.aspx
Cyprus	http://www.cylaw.org/
Czech Republic	http://zakony-online.cz/
Denmark	https://www.retsinformation.dk/
Estonia	https://www.riigiteataja.ee/
Finland	https://finlex.fi/fi/
France	https://www.legifrance.gouv.fr/
Germany	https://www.bgbl.de/
Greece	http://www.et.gr/
Hungary	http://mkogy.jogtar.hu/
Ireland	http://www.irishstatutebook.ie/
Italy	http://www.normattiva.it/
Latvia	https://likumi.lv/
Lithuania	https://www.e-tar.lt/
Luxembourg	http://legilux.public.lu/
Malta	http://justiceservices.gov.mt/
Netherlands	http://wetten.overheid.nl/
Poland	http://isap.sejm.gov.pl/
Portugal	http://www.pgdlisboa.pt/home.php
Romania	http://legislatie.just.ro/
Slovakia	https://www.slov-lex.sk/
Slovenia	http://www.pisrs.si/
Spain	http://www.boe.es/
Sweden	http://www.riksdagen.se/sv/dokument-lagar/

Supplementary Table 1. National legislation databases searched in review Phase 1. Sourced from Angers A, Bohacova A, Kaye J, et al. (2018) Overview of EU national legislation on genomics. URL: https://op.europa.eu/en/publication-detail/-/publication/503c92f7-ff55-11e8-a96d-01aa75ed71a1

**Who**	
	- Who is able to and/or typically provides genetic counselling services?<br>- Who is allowed to order (predictive/diagnostic) germline and somatic testing?<br>- Is ‘medical/clinical genetics’ a sub-specialization in the medical field?<br>- Approximately how many ‘geneticists’ are in <country>?<br>- Do genetic counsellors exist as a separate group of healthcare professionals?
**What**	
	- Is the content of genetic counselling sessions regulated or prescribed?<br>- How are genetic counselling sessions integrated into the clinical setting (i.e. flow of information and referrals)?<br>- How are literacy issues addressed for patients and/or non-geneticist clinicians?
**When**	
	- When are genetic counselling sessions typically provided?<br>- Are there any requirements and/or restrictions governing when genetic counselling sessions are delivered?<br>- Are clinician reimbursement frameworks a barrier to genetic counselling delivery?
**Where/How**	
	- Are telemedical genetic counselling sessions possible and/or used?<br>- If yes, can you estimate a rough proportion of in-person vs. telemedical genetic counselling?<br>- Are there any other requirements and/or restrictions governing where and how genetic counselling sessions can be delivered?

Supplementary Table 2. Guiding questions for semi-structured interview with expert representatives from each Member State.

		Where defined in legislation			
**Member State**	**National Legislation**	*Who*	*What*	*When*	**Additional Notes**
Austria	Gene Technology Act (1994)	Article 69, paragraph 1	Article 69, paragraphs 3-6	Article 69, paragraphs 3 & 4	-
Belgium	Agreement between the medical care insurance committee and the centres for human heredity for benefits in kind concerning genetic disorders: Genetic counselling, DNA tests carried out abroad (2011) – *implementing*: Law 14/7/1994 Compulsory Health Care and Indemnity Insurance Act, Article 22, 18°	Articles 10-14	Article 1, Sections 1-3	Article 1, section 2	Article 6 defines 'where' genetic counselling may be performed: Centre of Human Genetics, or hospital/care facility with formalized cooperation with Centre of Human Genetics
Bulgaria	Health Law (2005)	-	-	-	Article 140, paragraph 1 references specific informed consent provisions for genetic tests
Croatia	Law on the Protection of Patients' Rights (2008)	-	-	-	Article 22 references need for appropriate genetic counselling accompanying genetic tests
Czech Republic	Act 373/2011 Coll. on Specific Health Care Services (2011)	Article 28, paragraph 6	Article 28, paragraph 4a	Article 28, paragraph 6	-
Denmark	Amendment of the Healthcare Law, 27/01/2022	-	-	-	Section 29a references specific informed consent provisions for genetic tests
France	Law no. 2021-1017 of August 2, 2021 on Bioethics	Section 32	Sections 15 & 16	Sections 14 & 15	-
Germany	Gene Diagnostics Law (2009)	Section 7, paragraphs 1 & 3	Section 10, paragraph 3	Section 10, paragraphs 2 & 3	-

Hungary	Parliamentary Act No XXI	Section 3, definition m	Section 3, definition m	Section 6, paragraphs 2 & 4	-
Ireland	Disability Act (2005)	-	Section 42, paragraph 3	Section 42, paragraph 3	-
Latvia	Human Genome Research Act (2004)	-	-	-	Chapter II establishes principles of informed consent related to gene donors in genetic research
Luxembourg	Nomenclature Law (2014)	Section 10	Section 10	Section 10, paragraphs a-f & i	
Luxembourg	Hospital Law (2018)	-	-	-	Article 6, paragraph 3 specifies that genetic counselling is managed by the National Health Laboratory
Netherlands	Special Medical Procedures Act (2018)	Article 1.h	-	-	-
Portugal	Personal Genetic Information and Health Information Act (2005)	Article 9, sentences 2 & 3	-	Article 9, sentences 2 & 8	-
Slovenia	Criminal Code	-	-	Article 114, section 3	-
Spain	Law 14/2007, of 3 July, on Biomedical Research	-	Article 3, section e	Article 3, section e	-
Sweden	Genetic Integrity Act (2006)	-	-	-	Chapter 4, section 1 alludes to a procedure analogous to genetic counselling for prenatal diagnostics

Supplementary Table 3. Details of genetic counselling inclusion in national legislation in EU Member States.

Member State	National Regulation	Where defined in regulation<br>*Who*	*What*	*When*	Additional Notes
Austria	Quality Standard for Genetic Counselling and Diagnostics (2015)	Sections 3.1 & 3.2	Sections 3.1 & 3.2	Sections 3.1 & 3.2	-
Belgium	Royal Decree establishing the standards to be met by centers for human genetics (1987)	-	Article 6, paragraphs 1-3	-	--
Belgium	Ministerial Decree establishing special criteria for recognition of physician specialists, internship masters, and internship services in clinical genetics (2017)	Appendix 1.4	-	-	-
Bulgaria	Medical Standards for Medical Genetics (2011)	Article II, section 1	Article II, sections 4 & 5	Article II, sections 4 & 5	Section 2.1.1 also defines physical characteristics of office where genetic counselling can be conducted
France	Order of 27 May 2013 defining the rules of good practice applicable to the examination of a person's genetic characteristics for medical purposes	Section 2.2	Sections 3.1 & 3.2	Section 4.2	-
France	Decree no. 2022-1488 of November 29, 2022 on the conditions for prescribing certain medical biology examinations and communicating their results by genetic counsellors	Whole document	-	-	-
Hungary	Directive of the Ministry of Human Resources on genetic counselling, ID: 002092 (2020)	IV/1.	IV/1.	IV/1.	

Italy	General Authorisation No. 8/2014 for the Processing of Genetic Data (2014)	Sections 1h & 5.1	Section 1h	Section 5.1	-
Italy	Guidelines for Medical Genetics (2004)	Section 2	Section 2	Section 2	Section 5.2 specifies role for medical geneticists in improving genetic literacy of population
Latvia	Regulation No. 555, Adopted 28 August 2018 – Procedures for the Organization of and Payment for Health Care Service under Medical Treatment Law (1997)	-	-	Sections 74 – 75	-
Lithuania	Order of the Ministry of Health V-745 (2012)	Sections 5 & 17	-	-	Sections 7-12 also defines physical characteristics of office where genetic counselling can be conducted
Lithuania	Order of the Ministry of Health V-1458 (2014)	Section 3	-	Section 3	-
Netherlands	Planning Decision on Clinical Genetic Research and Genetic Counselling (1998)	Section 3.1	-	-	Section 2 restricts delivery of genetic counselling to 8 centres of clinical genetics; section 3.5 specifies role of medical genetics centres in educating non-genetics health professionals
Poland	Order of the Minister of Health of 21st July 2022 amending the Regulation on guaranteed benefits in the field of ambulatory care for families	Section 1	Section 1	Section 1	

	with high risk of hereditary cancer				
Portugal	Order of the Ministry of Health #5411/97 (1997)	Section 3	-	-	-
Romania	Establishment of the Medical Genetics Network (2014)	Annex 3	-	-	Annex 4 specifies health education for professionals and the public as regional medical centre duty
	Order no. 774 of September 18, 2023	-	-	-	Introduces National Genetic Testing Subprogram, broadly improving clinical integration of genetics
	Appendices no. 1 and 2 to Government Decision no. 521/2023 for approval of the service packages and the framework contract that regulates the conditions for the provision of medical assistance, medicines and medical devices, within the social health insurance system	Chapter II, Section B 1.8	Chapter II, section B 1.7	-	Section B 1.7 and 1.8 specify that genetic counseling is reimbursed as a service in outpatient care. Psychological counselling can also be added to genetic counselling (as connected services)
Slovakia	The Concept of Healthcare in the Field of Medical Genetics (2014)	Whole document	Section 1.2.4	Section 1.2.4	Sections 1.1 & 2.3 specify role of medical genetics departments to provide health education of the population by medical genetics departments; Section 3 extends this directive to the continuing education of other health workers

Supplementary Table 4. Details of genetic counselling inclusion in national regulations in EU Member States.

Country	Who: Provides genetic counselling	Who: Allowed to order germline tests	Who: Is genetics a clinical specialty?	Who: Are genetic counsellors recognized?	What: Guides counselling content	When: Is genetic counselling provided	Where/How: Telemedical counselling	Where/How: Frequency of telemedical counselling	**Reimbursement barriers to counselling**
Austria	Typically, medical geneticists; possible by any specialized medical doctor in his/her field	Any specialized medical doctor for tests related to his/ her field	Yes	No	National Legislation & Regulation	Mandatory pre- and post-test	Possible	<5-10% of all counselling	Counselling only reimbursed when delivered by centers of medical genetics; no reimbursement for individual doctors
Belgium	Covered by insurance convention (most scenarios): multidisciplinary team (including clinical geneticists, psychologists, social workers, and genetic counsellors); possible by all physicians/ affiliated genetic counsellors in scenarios outside convention	Any medical doctor	Yes	No (*but well-integrated into genetic services delivery*)	Convention between insurance and clinical genetics centers; clinical best practice	Mandatory pre- and post-test (most scenarios); Typically pre- and post-test (scenarios outside convention)	Possible	<5% of all counselling	Counselling only full reimbursed when covered under convention; 12 EUR per consult co-pay for counselling performed outside convention (e.g. mainstreaming)
Bulgaria	Medical geneticists	Medical geneticists & oncologists	Yes	No	National clinical practice standards (*regulation*)	Mandatory pre- & post-test	Possible	'Relatively frequent'	National insurance doesn't reimburse for counselling; provided for free or paid by patient in public system
Croatia	Any medical doctor, but mostly pediatricians with	Any medical doctor	Yes (*1st intake of medical*	No	Clinical best practice	Typically pre- & post-test	Possible	Minority of counselling sessions	None

	genetics sub-specialization (*medical genetics new specialty, 1st residents in 2023*)		*genetics residents in 2023*)						
Cyprus	Typically, genetic counsellors; possible by any medical doctor	Any medical doctor	Yes	No (*but well-integrated into genetic services delivery*)	Clinical best practice	Typically pre- and post-test	Not possible	-	None
Czech Republic	Medical geneticists	Medical geneticists	Yes	No	National legislation; clinical best practice	Mandatory pre- and post-test	Possible	Approx. 20% of all counselling	None
Denmark	Smaller panels: any clinician + nurses; WGS: medical geneticist	Smaller panels: any clinician + nurses; WGS: medical geneticist	Yes	No	Principles of informed consent	Mandatory pre-test only; post-test possible	Possible	'Quite frequent'	None
Estonia	Medical geneticists; some aspects provided by nurses in specific scenarios	Any medical doctor	Yes	No	Clinical best practice	Typically pre- and post-test	Possible – for recurrent visits	Minority of consults, but growing in proportion	Lump sum amount to cover all genetic services for entire year; non-priority cases at end of year typically asked to wait until next year
Finland	Typically medical geneticists, possible by any medical doctor; 'unwritten rule' that medical geneticists handle predictive testing; in certain situations, nurses with training in genetics	Any medical doctor	Yes	No (*but nurses with training in genetics integrated into genetic services delivery*)	Clinical best practice; principles of informed consent	Typically pre- & post-test	Possible	>50% of all counselling	None

France	Predictive: only medical geneticists or genetic counsellors; Symptomatic: any medical doctor with additional genetics training	Predictive: only medical geneticists or genetic counsellors; Symptomatic: any medical doctor with additional genetics training	Yes	Yes	National legislation & regulation	Mandatory pre- & post-test	Possible	Approx. 10% of all counselling	National insurance reimbursement only ~50% of costs of prescribed genetic tests and counselling; hospital required to cover other ~50%
Germany	Clinical geneticists or specialist doctors with additional genetics qualification in field of primary expertise	Diagnostic scenario: any medical doctor; predictive scenario: clinical geneticists or specialist doctors with additional genetics qualification in field of primary expertise	Yes	No (*but sparsely working in health system)*	National legislation	Typically pre- & post-test	Possible	Rarely used	No reimbursement of telemedical counselling
Greece	Any medical doctor	Any medical doctor	Yes	No	Clinical best practice for breadth of specialties	Variable; typically pre-test	Possible	Used sparingly	Inconsistent and insufficient reimbursement of both counselling and testing
Hungary	Clinical geneticists (*results cannot be withheld by any specialist, but genetic counselling can only be provided by a clinical geneticist*)	Typically clinical geneticists, but possible by any specialist medical doctor in cases of urgency or common indications covered by their fields of expertise (*with access to a clinical genetics consultation at all times*)	Yes	No	National legislation; national directive on genetic counselling(58)	Mandatory pre- & post-test	Possible	Used sparingly, <10% of all counselling; upon request of counselee	Insurance only reimburses 1 test/ consultation per patient per year and only selected tests

Ireland	If known diagnosis, typically genetic counsellors; if unknown diagnosis, typically clinical geneticists; also open to other specialties	Any medical doctor *(but currently limited to certain hospitals)*	Yes	No *(but working in health system)*	Clinical best practice	Typically pre- & post-test	Possible	Somewhat frequent	Dedicated budget for genetic testing (*and associated counselling*) only at one hospital; testing at other hospitals must be sourced from general hospital budget
Italy	Clinical geneticists, oncologists; in specific circumstances laboratory scientists with medical genetics specialization	Clinical geneticists, oncologists	Yes	No	National regulation	Mandatory pre- & post-test	Possible	Minority of consults	None
Latvia	Medical geneticists	Single mutation diagnostics: 14 specialties; WES: Medical geneticists & pediatric oncologists	Yes	No	Clinical best practice	Mandatory pre- & post-test	Possible	Used sparingly to deliver negative test results	Counselling restricted by available funds for genetic testing
Lithuania	Clinical geneticists; OBGYN for pre-natal testing	Clinical geneticists	Yes	No	Clinical best practice	Typically Pre & post-test	Possible	Approx. 60% of all counselling	None
Luxembourg	Clinical geneticists	Clinical geneticists/ any medical doctor for tests related to his/her field	Yes (*but no in-country training*)	No	National legislation for reimbursement; clinical best practice	Typically pre- & post-test	Not possible	-	Patients first must cover the bill and are then reimbursed by national insurance only 88% (100% for children <18 years); genetic counselling only reimbursed when prescribed by specialized medical doctors, not

									generalists; no reimbursement of telemedical counselling
Malta	Primarily clinical geneticists; also ‘genomic care coordinators’ (analogous to genetic counsellors)	Primarily clinical geneticists	Yes	No (*but process for recognition ongoing*)	Clinical best practice	Typically pre- & post-test	Possible	Rather frequent	None
Netherlands	Clinical geneticists; also specifically trained specialist nurses and physician assistants	Clinical geneticists and specifically trained specialist nurses and physician assistants	Yes	No	Standard operating procedures in each clinical genetics center*	Mandatory pre- & post-test	Possible	Rather frequent	None for clinicians; patients may first have to cover annual excess (up to 385 EUR) before insurance active
Poland	Primarily clinical geneticists; open to all physicians	Any medical doctor	Yes	No (*but planned in upcoming legislation*)	Clinical best practice	Typically pre- & post-test	Possible	Minority of consults	None
Portugal	Any medical doctor	Predictive – only medical geneticists; otherwise any medical doctor	Yes	No (*exist but not recognized or integrated*)	National protocol for pre-symptomatic testing for incurable late-onset diseases; otherwise clinical best practice	Variable	Possible	Minority of consults	None
Romania	Clinical geneticists	Clinical geneticists	Yes	No	Clinical best practice	Typically pre- & post-test	Possible	Minority of consults	Genetic counselling covered by insurance, but genetic testing reimbursed by

									mosaic of health ministry and private funding
Slovakia	Medical geneticists	Medical geneticists	Yes	No	National regulation; clinical best practice	Typically pre- & post-test	Possible	Used sparingly	None for Dept. of Medical Genetics, but reduced for telemedicine
Slovenia	Mainly clinical geneticists; ‘genetic nurses’ (nurse with additional genetics training) also included; possible by any medical doctor	Open to all specialties; mainly clinical geneticists	Yes	No	Local standard operating procedures	Mandatory pre- & post-test	Possible	Used sparingly	None
Spain	Specialist medical doctor, mainly medical oncologists/ gastroenterologists for hereditary cancer; in Catalonia, genetic counsellors ‘unofficially’	Open to all specialties; mainly medical oncologists and gastroenterologists for hereditary cancer	No	No (*practicing in Catalonia, but not recognized*)	National legislation; clinical best practice	Mandatory pre- & post-test	Possible	Minority of consults	None
Sweden	Any medical doctor can provide; medical geneticists/genetic counsellors provide for complex cases and in case of positive test results	Any medical doctor	Yes	No (*practicing but not formally recognized*)	Clinical best practice	Typically pre- & post-test	Possible	Frequently used	Generally none, but can be some regional variation; reimbursement subject to regional agreements

Supplementary Table 5. Overview of genetic counselling practice in EU Member States, as reported in interviews with national expert representatives from each country. ‘WGS’ = whole genome sequencing. ‘WES’ = whole exome sequencing. ‘Medical geneticists’ and ‘clinical geneticists’ are functionally equivalent labels given in different countries to describe physicians specialized in genetics. * - includes 1) guidelines and 4-year education program to become a clinical geneticist after medical (MD) training; 2) guidelines for specific genetic conditions; and 3) guidelines for mainstreaming.
